# Data Quality and Reliability Assessment of Wearable EMG and IMU Sensor for Construction Activity Recognition

**DOI:** 10.3390/s20185264

**Published:** 2020-09-15

**Authors:** Srikanth Sagar Bangaru, Chao Wang, Fereydoun Aghazadeh

**Affiliations:** 1 Bert S. Turner Department of Construction Management, Louisiana State University, 237 Electrical Engineering Building, Baton Rouge, LA 70803, USA; sbanga3@lsu.edu; 2 Bert S. Turner Department of Construction Management, Louisiana State University, 3315D Patrick F. Taylor Hall, Baton Rouge, LA 70803, USA; 3 Department of Mechanical & Industrial Engineering, Louisiana State University, 3250A Patrick F. Taylor Hall, Baton Rouge, LA 70803, USA; aghazadeh@lsu.edu

**Keywords:** wearable sensor, reliability, construction activity classification, electromyography, inertial measurement unit, data quality

## Abstract

The workforce shortage is one of the significant problems in the construction industry. To overcome the challenges due to workforce shortage, various researchers have proposed wearable sensor-based systems in the area of construction safety and health. Although sensors provide rich and detailed information, not all sensors can be used for construction applications. This study evaluates the data quality and reliability of forearm electromyography (EMG) and inertial measurement unit (IMU) of armband sensors for construction activity classification. To achieve the proposed objective, the forearm EMG and IMU data collected from eight participants while performing construction activities such as screwing, wrenching, lifting, and carrying on two different days were used to analyze the data quality and reliability for activity recognition through seven different experiments. The results of these experiments show that the armband sensor data quality is comparable to the conventional EMG and IMU sensors with excellent relative and absolute reliability between trials for all the five activities. The activity classification results were highly reliable, with minimal change in classification accuracies for both the days. Moreover, the results conclude that the combined EMG and IMU models classify activities with higher accuracies compared to individual sensor models.

## 1. Introduction

The construction industry is one of the leading industries in the world, which spends $10 trillion on construction-related goods and services every year [1]. However, the construction industry is facing a massive workforce shortage of skilled craft workers [2]. More than 8 out of 10 construction firms report having a hard time finding qualified workers. One of the significant causes of workforce shortage is the premature retirement of skilled craft workers due to safety and health issues. Due to a lack of proper safety training and monitoring systems, the construction workforce is exposed to various fatal and non-fatal injuries such as work-related musculoskeletal disorders (WMSDs). To overcome these challenges, various researchers have proposed wearable sensor-based systems in the area of construction safety and health [3,4,5,6,7,8]. Various applications in the area of safety and health involve preventing musculoskeletal disorders, fall prevention, mental and physical workload assessment, and fatigue monitoring [3,4,5,6,7,8]. All these applications can be categorized as a classification problem since they involve identifying different postures, classifying different physical and mental workloads, or detecting different motions or gestures using the sensor data. Moreover, classifying workers’ activity helps in monitoring and managing the productivity, safety, and quality of work [9].

In the construction domain, the wearable sensor-based activity recognition models have gained increased attention due to low-cost, ease of use, high accuracy, and non-intrusiveness. Most of the previous studies have used accelerometers and gyroscopes embedded in smartphones to recognize construction workers’ activity [10,11,12,13]. A study by Cezar [14] used an accelerometer and gyroscope embedded in the smartphone placed on the dominant hand to recognize hammering, sawing, sweeping, and drilling activities with an accuracy of 91% using quadratic discriminant analysis (QDA) algorithm. Lim, et al. [15] and Akhavian and Behzadan [16] have developed artificial neural network (ANN) based models for identifying falls and manual material handling activities with an accuracy of 94% and 90.74% using the smartphone placed in the hip pocket and upper arm respectively. The ironwork activities recognition models developed by [17] and [9] using support vector machine (SVM) and decision trees (DT) were able to recognize activities with 94.83% and 92.98% accuracy. Even though these smartphone sensors-based models have achieved considerable accuracy, there are practical implementation challenges. In order to overcome the smartphone challenges, inertial measurement unit (IMU), sensor-based activity recognition models have been proposed for various construction applications such as work sampling [18,19,20] and fall detection [21]. The wearable IMU sensor-based models have used various machine learning algorithms such as DT, random forest, and SVM to recognize ironwork [18], fall detection [19], and bricklaying [20] with an accuracy of 90.4%, 93.90%, and 88.1% respectively. However, the current activity classification methods are that they are limited to a fewer number of activities involving either upper body or lower body, use of multiple sensors, and do not consider activities with multiple intensities. Moreover, none of these studies have discussed the reliability of sensor data and classification results. Therefore, there is a necessity for low-cost, easy to use, and non-obstructive sensors that can provide reliable data for complex construction activity classification.

Despite the fact that sensors provide rich and detailed information, not all sensors can be used for construction applications due to the dynamic nature of construction work [3]. It was recommended that multisensory data fusion, which was applied in other domains, provides an opportunity for enhancing the accuracy of activity classification [6,22]. The sensor for construction applications should be simple and easy to wear, unobtrusive, affordable, and wireless. Moreover, the sensor should provide reliable data and involve minimal or no preprocessing for noise removal. Therefore, it is essential to identify a suitable and reliable sensor for construction activity classification, which helps in developing construction workers’ safety and health monitoring systems to prevent work-related injuries such as WMSDs, which is one of the significant reasons for the workforce shortage.

The armband sensor is an affordable, non-invasive, lightweight, and wireless wearable armband sensor that is available off-the-shelf to collect workers’ forearm electromyography (EMG) and inertial measurement unit (IMU) data [23]. Many researchers have used these signal data for different applications in various domains. To the best of the authors’ knowledge, none of the studies in the construction domain have explored the use of armbands and a combination of EMG and IMU data for construction applications. Furthermore, investigating muscle activity and kinematic signals provides an understanding of workers’ physiological responses to workload. Furthermore, the signals facilitate activity analysis and workers’ behavior towards the work. In order to choose a wearable sensor for any construction applications specifically for activity classification, it is essential to investigate the data quality and reliability because the muscle activity and motion sensor signals from the forearm may inevitably be contaminated due to noise signals and artifacts that originate at the skin-electrode interface or due to external sources. A reduction in the noise and artifact contamination is required as well as preservation of the required information from the signals. Moreover, the sensor should provide consistent and reliable signals for activity throughout the data collection process. Therefore, the objective of this study is to assess the data quality and reliability of forearm EMG and IMU data for construction activity classification by following the guidelines, recommendations, and methods for data quality and reliability assessment proposed by previous studies on sensor [24,25,26,27,28,29].

In order to achieve the proposed objective, the whole study is divided into seven experiments. The first three experiments involve evaluating the data quality, understanding the effect of armband position on data quality, and reliability of forearm EMG and IMU data. Later, four experiments involve building and evaluating activity classification models, assessing the reliability of classification results, understanding the effect of lifting weights on classification results, and evaluating the classification performance of different sensor combinations. The results of these experiments answer various questions such as noise level in armband signal data, drift in the IMU sensor data, quality of EMG and IMU data for at-rest and in-motion activities, the effect of armband position on signal quality, the accuracy of construction activity classification using EMG and IMU, reliability of sensor data and classification results, effect of lifting weights on classification accuracy, and classification performance of different sensor combinations. It was hypothesized that the armband sensor provides reliable EMG and IMU data and activity classification results. The answers to the above questions establish the reliability and applicability of forearm EMG and IMU data for construction activity classification.

## 2. Materials and Methods

### 2.1. Participants

Eight healthy college male students voluntarily participated in all the experiments. The participants’ ages ranged from 24 to 28 years (mean ± SD: 26.13 ± 1.55 years), height ranged from 1.65 to 1.83 m (1.74 ± 0.06 m), and weight ranged from 62.60 to 100 kg (81.35 ± 12.44 kg). All the participants were right-handed, healthy, and had no musculoskeletal disorders at the time of experiments. All the procedures involving human participants were approved by the Louisiana State University Institutional Review Board (IRB #: IRBAM-20-0112). The purpose of the research was demonstrated to all the participants before the start of the experiment, and their signatures were obtained on the informed consent forms. The sample size required to assess the reliability of the sensor using the intraclass correlation (ICC) was determined using the tables from Bujang and Baharum [30]. An ICC score greater than or equal to 0.75 indicates excellent reliability [31,32]. At least seven participants are required to achieve a minimum of 0.75 ICC scores with two assessments per subject at a 0.05 significance level and a power of 0.80 [30].

### 2.2. Measurements and Instrumentation

**Figure 1:**
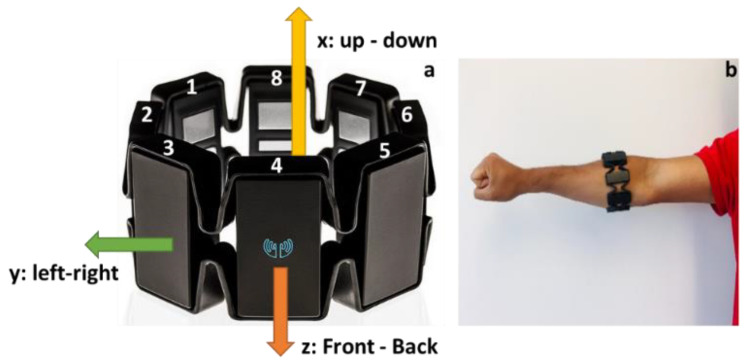
(**a**) Myo armband electrode location and (**b**) Myo armband placement on the forearm.

A forearm based wearable armband sensor (Myo armband) developed by Thalmic Labs Inc. was used to collect the EMG and IMU data. Myo armband sensor is a non-intrusive wearable sensor that consists of eight dry surface EMG sensors and a 9-axes IMU sensor (3-axes gyroscope, 3-axes accelerometer, and 3-axes magnetometer). The sensor weighs approximately 93 g [23]. The data from the sensor is transmitted to the computer or cloud storage via Bluetooth Low Energy (BLE) wireless connection. The raw EMG and IMU data can be assessed through the Myo software development kit (SDK). The Myo SDK was used to acquire real-time forearm EMG and IMU data at a frequency of 200 Hz and 50 Hz, respectively. The device goes into an idle state if there is no activity for more than 30 s. The configuration of Myo armband electrodes is shown in Figure 1a, where the electrode with the LED light and Myo logo is channel-4, followed by channel-3 in clockwise direction and channel-5 in counter-clockwise direction. Moreover, Figure 1a shows the direction of x, y, and z of the IMU sensor. The armband was worn on the thickest part of the forearm, as shown in Figure 1b with the channel-4 in the line of the index finger, and the blue marker was in the lower forearm for the experiments unless otherwise stated [33]. After wearing the armband sensor, the participant calibrates their motion by performing predefined gestures such as finger spread, wave-in, wave-out, and relaxed state gestures by connecting with Thalmic Labs’ Myo Connect manager [34].

The eight EMG sensors capture the electrical impulses generated by the forearm muscles, which are returned as an 8-bit array, in other words, each EMG sensor outputs an integer value between −128 and 127 representing muscle activation levels. The armband sensor captures the muscle activity of various forearm muscles such as the brachioradialis, flexor digitorum superficialis, medial epicondyle of humerus, palmaris longus, flexor carpi ulnaris, flexor carpi radialis, and pronator teres [35]. Whereas, the IMU unit captures the motion of the forearm by measuring acceleration, angular velocity, and orientation along the x, y, and z axes. It was ensured that the armband was always synced with the application and calibrated throughout the experiments.

High-precision conventional wearable EMG and IMU sensors such as FREEEMG (BTS Bioengineering Corp., Quincy, MA, USA) and YEI 3-Space IMU sensor (Yost Engineering Inc., Portsmouth, OH, USA) respectively, were used to compare the armband sensor data quality. The conventional sensor measures the acceleration and gyroscope in units of g and radians/s, respectively. In comparison, the conventional EMG sensor measures muscle activity in millivolts (mV). Besides, the conventional IMU sensor was calibrated using a gradient descent calibration procedure and no preprocessing was performed on any of the sensor data before data quality calculations.

To assess the reliability of the armband sensor data, features such as absolute acceleration, absolute angular velocity, and mean absolute value of EMGsum (sum of EMG values) were calculated from raw data [36]. These sensor features are widely used in activity/gesture/motion recognition applications [33,36,37,38,39,40,41,42]. The acceleration along x, y, and z axes were used to compute the absolute acceleration or magnitude of the acceleration vector (Acc) at any given timestamp (t) using Equation (1) [31,43,44]. Similarly, the angular velocity along the three axes provided by the gyroscope sensor was used to calculate the absolute gyroscope angular velocity or magnitude of gyroscope vector (Gyro) at any given timestamp (t) using Equation (2) [31,44,45,46]. For simplicity, the angular velocity along the axes was represented as Gyro in Equation (2). Using the eight EMG values, a new feature EMGsum was calculated by summing up all the eight EMG values at any timestamp (t) [47,48]. Further, the mean absolute value (MAV) of EMGsum was evaluated using Equation (3), which was later used for reliability assessment [33,37,38]. For each trial, an average of acceleration magnitude, an average of gyroscope magnitude, and MAV of EMGsum was computed to assess the trial-to-trial (intra-day) reliability of the sensor. Whereas in the case of day-to-day (inter-day) reliability test, the mean values of three trials of each day were used for ICC analysis.

(1) $$\mathrm{Acc}(t)=\sqrt[{2}]{{\mathrm{Acc}(t)}_{x}^{2}{+\mathrm{Acc}(t)}_{y}^{2}{+\mathrm{Acc}(t)}_{z}^{2}}$$

(2) $$\mathrm{Gyro}(t)=\sqrt[{2}]{{\mathrm{Gyro}(t)}_{x}^{2}{+\mathrm{Gyro}(t)}_{y}^{2}{+\mathrm{Gyro}(t)}_{z}^{2}}$$

(3) $$\mathrm{MAV}=\frac{1}{N}\overset{N}{\underset{k=1}{\sum }}|{\mathrm{EMGsum}}_{k}|$$

### 2.3. General Procedures of the Study

This study consists of seven experiments, including (a) evaluating the forearm EMG and IMU data quality for “at-rest” and “in- motion” activities (Experiment I); (b) investigating the effect of armband sensor position on EMG and IMU data (Experiment II); (c) assessing the reliability of forearm EMG and IMU data obtained while performing construction activities (Experiment III); (d) classification model building, performance evaluation, and classifier comparison (Experiment IV); (e) investigating the reliability of results obtained from classification models using EMG and IMU data while performing construction activities on different days (Experiment V); (f) investigating the effect of lifting weight on forearm EMG and IMU data and activity classification results (Experiment VI); and (g) comparison of activity classification performance for different sensor combinations. The activities performed by the participants are standardized across all the experiments. The “at-rest” activities include the armband lying stationary on the floor or placed on the arm of a person sitting still with arm resting on a desk. Whereas, the “in- motion” activities include screwing at elbow height at a frequency of 1 turn/6 s, wrenching while kneeling at a frequency of 1 turn/6 s, lifting a 25 lbs sandbag from elbow to shoulder height at a frequency of 1 lift/6 s, and carrying a 25 lbs sandbag on the shoulder with the dominant hand at the bottom of the sandbag for 30 s. Activities were designed in such a way that they represent a wide range of construction activities involving forearm (lifting), wrist (screwing and wrenching), and whole-body (carrying). Moreover, these activities represent controlled natural motions such as repeated motion (lifting), impulsive motion (screwing or wrenching), and free motion (carrying). All the activities were performed for 30 s (i.e., each trial of activity was 30 s). Each participant performed three trials for an activity on a testing day. There were two testing periods (i.e., Day-1 and Day-2) where participants performed all five activities (i.e., stationary on the body, screwing, wrenching, lifting, and carrying) on both days. Therefore, each participant performed a total of 15 activities (3 trials × 5 activities) in one day. There was no gap between the testing periods. The activities were randomized for all the participants for both days. Before the start of the experiment, all the participants were given enough time to familiarize themselves with the tools to eliminate systematic bias, which occurs due to learning effects [49]. The participants were asked to warm up their bodies before the start of the session, and enough rest was provided between the trials to prevent injuries and fatigue [50]. Once the armband was worn on the body and synced with the computer, a two minute settling time was considered before the start of the experiment to prevent the rotational drift. In order to test the reliability using the test-retest approach, all the activities were performed in an indoor environment under control conditions unless stated otherwise. The eight participants’ EMG, accelerometer, and gyroscope data were recorded and stored for all five activities for both the days. The data were processed and analyzed accordingly based on the experiment requirements. The seven experiments mentioned above are further explained in the following sections and broadly divided into three categories: data quality assessment, data reliability assessment, and activity classification performance evaluation.

#### 2.3.1. Data Quality Assessment

##### Experiment I—Evaluating the Forearm EMG and IMU Data Quality for “At-Rest” and “In-Motion” Activities

**Figure 2:**
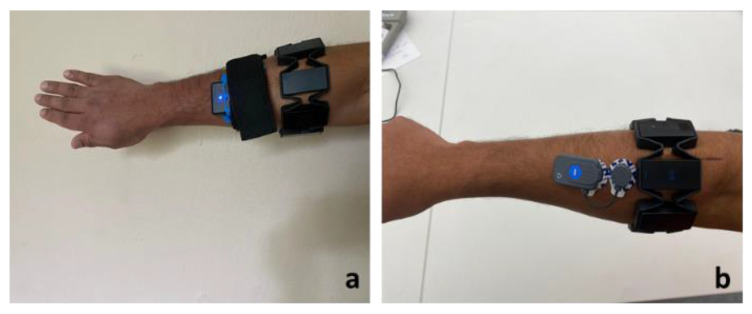
Position of conventional (**a**) inertial measurement unit IMU and (**b**) electromyography (EMG) along with armband sensor.

The wearable sensor data is highly susceptible to various confounding factors that affect the quality of data. In this experiment, the data quality of EMG, acceleration, and gyroscope measurements were assessed by evaluating the signal-to-noise ratio (SNR) and compared to a conventional sensor. Furthermore, the influence of confounding factors (communication devices, another sensor, power tools, and smartwatches) and environments (indoor and outdoor) on the data quality were studied in this experiment. Firstly, the data quality was determined for the armband sensor and compared with the conventional sensors for at-rest and in-motion activities. In order to compare the data quality of the armband sensor, the conventional sensors were placed along with the armband sensor while performing activities, as shown in Figure 2. Each in-motion activity was performed three times by all eight participants. The average SNR value was used for the comparison. The influence of various confounding factors and environmental conditions on the armband sensor data quality was assessed when Myo was lying on the floor by computing SNR values for three trials. Inter-device data quality was assessed using two armbands lying on the floor at the same time to check if the data is consistent across different devices under the same conditions. All the at-rest activities were conducted three times, and the average value was considered to represent the influence of confounding factors, environment, and inter-device variability on the data quality.

##### Experiment II—Investigating the Effect of Armband Sensor Position on EMG and IMU Data

**Figure 3:**
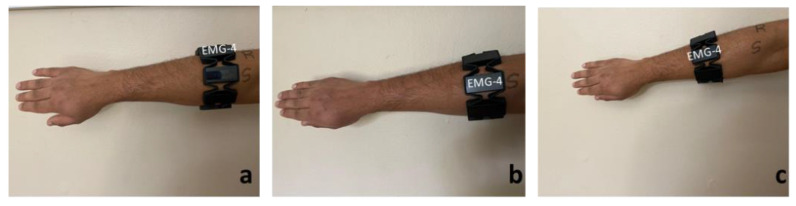
Three armband sensor positions to test the effect of sensor position on data quality: (**a**) rotated, (**b**) standard, and (**c**) slid down.

In order to explore the effect of sensor position on the EMG and IMU data, a lifting activity was performed for three different sensor positions as shown in Figure 3. The standard position refers to wearing an armband with sensor-4 in the direction of the index finger. Whereas the rotated and slid positions refer to rotating the armband in an anticlockwise direction (sensor-5 in the direction of the index finger) and sliding the armband downwards with respect to the standard position, respectively. A qualitative analysis was performed on the root mean square value of EMG and the absolute magnitude of IMU data collected while performing lifting activity with three sensor positions.

The sensor data quality was assessed by evaluating the noise level in the data using the signal-to-noise ratio (SNR). The SNR value of a signal is the ratio of the power of the signal to the power of the noise [51]. Alternatively, it is defined as the ratio of the mean of the measurements (µ) to the standard deviation of the measurements (σ) as shown in Equation (4). Where mean and standard deviation (SD) of measurements represent the power of signal and power of noise in the measurements. The signal power of acceleration and gyroscope measurements were determined as mean values of absolute magnitude. Whereas, the mean value of the EMG measurements was calculated as mean-absolute-value (MAV) [25,52].

(4) $$\mathrm{SNR}=\frac{µ}{\sigma }$$

#### 2.3.2. Data Reliability Assessment

##### Experiment III—Assessing the Reliability of Forearm EMG and IMU Data Obtained While Performing Construction Activities

The EMG and IMU data collected from eight participants while performing construction activities on two different days were assessed for reliability. In this experiment, the raw EMG and IMU data collected from eight participants were processed to calculate the mean absolute value (MAV) of EMG, absolute acceleration (Acc), and absolute gyroscope (Gyro) for each trial of both the days (Day-1 and Day-2). The MAV, Acc, and Gyro values of each trial were used to assess trial-to-trial reliability for both the days. Further, the MAV, Acc, and Gyro values of all the three trials were averaged for an activity for a participant for each day to evaluate reliability between days. The relative reliability was assessed using the intraclass correlation coefficient, and absolute reliability was evaluated using standard error of measurement (SEM) and smallest detectable difference (SDD).

All statistical analyses were performed using IBM SPSS statistical package version 25 (Armonk, NY, USA). The trial-to-trial and day-to-day reliability were assessed on the accelerometer, gyroscope, and electromyography measurements obtained while performing five construction activities on two different testing periods (i.e., Day-1 and Day-2). Moreover, the assessment of the trial-to-trial and day-to-day reliability measures intradevice reliability. The reliability was assessed between the trials and between the days using test-retest reliability, which consists of relative and absolute reliability [31,53]. The relative reliability refers to the magnitude of the correlation of repeated measurements, which was evaluated using the intraclass correlation coefficient (ICC) [31,32]. The relative reliability was expressed using ICC form (3, k), which includes a two-way mixed effect model, mean of k measurement type, and a definition of a relationship as absolute agreement [54,55]. Moreover, the ICC form (3, k) considers both systematic and random errors and uses the mean value of the repeated measurements as evaluation scores [31]. Based on the ICC score, the strength of relative reliability can be interpreted as excellent (if ICC score is higher than 0.75), good (if ICC score is between 0.59 and 0.75), fair (if ICC score is between 0.48 and 0.58), and poor (if ICC score is less than 0.40) [31,32,54,55,56,57].

Whereas the absolute reliability refers to variability in the repeated measurements of an individual [31,32]. The absolute reliability was evaluated by estimating the standard measurement error (SEM). SEM estimates how the repeated measures of an individual on the same device tend to distribute around true value [31]. SEM is estimated as defined in Equation (5), where SD is the standard deviation of the measurements of a test and retest of all participants, and ICC is the average trial-to-trial or day-to-day test-retest relative reliability [31,32,56,58,59]. The SEM% was used to compare the absolute test-retest reliabilities of different scenarios, which was evaluated using Equation (6), where the SEM score is represented as a percentage of SEM divided by the mean of test and retest measurements. The SEM% value below 10% indicates excellent absolute test-retest reliability. Moreover, the smallest detectable difference (SDD) was calculated from SEM at a 95% confidence interval using Equation (7), which is the smallest change in the measurement that is required to be considered as a real change in the measurement but not due to error [31,32,56]. Similar to SEM%, the SDD score is expressed as a percentage of the mean of measurements (SDD%), which is computed using Equation (8) [31,32,56]. Before performing the parametric reliability testing, a nonparametric Kolmogorov–Smirnov test was performed to verify the normality of the data.

(5) $$\mathrm{SEM}=\mathrm{SD}\sqrt{1-\mathrm{ICC}}$$

(6) $$\mathrm{SEM}\%=\frac{\mathrm{SD}\sqrt{1-\mathrm{ICC}}}{\mathrm{Mean}}\times 100$$

(7) $$\mathrm{SDD}=1.96\times \sqrt{2}\times \mathrm{SEM}$$

(8) $$\mathrm{SDD}\%=\frac{1.96\times \sqrt{2}\times \mathrm{SEM}}{\mathrm{Mean}}\times 100$$

#### 2.3.3. Activity Classification, Performance Evaluation, and Classification Reliability

##### Experiment IV—Classification Model Building, Performance Evaluation, and Classifier Comparison

The data obtained from the armband sensor worn by the eight participants performing five activities on two different days (Day-1 and Day-2) was used to build machine learning (ML) based classifiers for respective days. A typical machine learning methodology, which includes data preparation, model building, model training, hyperparameter tuning, and model evaluation, was used to develop ML classifiers for activity classification, which was implemented using the PyCatet classification module in Google Colab. Firstly, the dataset was prepared using the raw acceleration (a_x_, a_y_, a_z_), gyroscope (g_x_, g_y_, g_z_), and EMG (8-channel) features for both the days. The 8-channel EMG data were downsampled by converting 8-bit to 32-bit to match the frequency of accelerometer and gyroscope data. Therefore, the final dataset for each day consists of 38 (3-acceleration, 3-gyroscope, and 32-EMG) input features. Further, the data were manually labeled for five different activities (i.e., stationary on the body, screwing, wrenching, lifting, and carrying). Once the datasets were prepared, the labeled data was used to build the machine learning (ML) based classifier models using the default classifier settings. Besides, the hyperparameters of the model were tuned by optimizing the model accuracy to obtain a finely tuned model. The ten most common ML-based classifier models such as random forest, J48 decision trees, support vector machine (SVM), naïve Bayes, k-nearest neighbors (KNN), logistic, multi-layer perceptron (MLP), linear discriminant analysis (LDA), quadratic discriminant analysis (QDA), and gradient boosting (Xgboost) were built using each day dataset. Additionally, 10-fold cross-validation was performed to evaluate the performance of the classifiers. In the cross-validation technique, the dataset is randomly shuffled and divided into ten groups. Each unique group is considered as a holdout or test dataset, and the remaining nine groups are used for model training. Once the model has been fitted on the training dataset, the model is evaluated on the test set. The evaluation score is retained, and the model is discarded. This process is repeated for each unique group. The performance of the trained ML classifier was evaluated using metrics such as accuracy, recall, precision, F1 score, kappa, and confusion matrix. The performance of different classifiers was compared to determine the best performing classifier for each dataset.

##### Experiment V—Investigating the Reliability of Results Obtained from Classification Models Using EMG and IMU Data While Performing Construction Activities on Different Days

The reliability of results obtained from the classification models using Day-1 and Day-2 datasets was investigated. The best classifier obtained in Experiment III was further used to run ten iterations on each dataset. The accuracies of the classifier on the Day-1 dataset were compared to accuracies of the same classifier on the Day-2 dataset using paired *t*-test at 0.05 significance level.

##### Experiment VI—Investigating the Effect of Lifting Weight on Forearm EMG and IMU Data and Activity Classification

Detecting different weights is useful for many construction applications. This experiment investigates if the weight affects forearm EMG and IMU data and activity classification. For this experiment, an activity of lifting three different weights (10 lbs, 25 lbs, 50 lbs) with three trials from four participants was considered. The raw data with 38 features (acceleration-3, gyroscope-3, and EMG-32) was manually labeled for three activities (Lift10, Lift25, and Lift50). The ML-based classification models such as random forest, J48 decision trees, support vector machine (SVM), naïve Bayes, k-nearest neighbors (KNN), logistic, multi-layer perceptron (MLP), linear discriminant analysis (LDA), quadratic discriminant analysis (QDA), and gradient boosting (Xgboost) were built using the raw data and evaluated using 10-fold cross-validation technique. The best classifier results were analyzed for three different classes to check if the sensor data could classify different weights.

##### Experiment VII—Comparison of Activity Classification Performance for Different Sensor Combinations

This experiment focuses on comparing the performance of various ML-based classifier models built using different sensor feature combinations such as EMG + IMU, IMU alone, and EMG alone. For this analysis, two datasets were considered, namely, controlled and uncontrolled activity datasets. The controlled activity dataset was prepared by combining the Day-1 and Day-2 data of five controlled activities, namely screwing, wrenching, lifting, and carrying. Whereas the uncontrolled dataset was prepared by collecting forearm armband data from the participants while performing nine construction activities at varied intensities and pace such as walking at random speed (walk), carrying (10 lbs, 25 lbs, and 50 lbs), lifting (10 lbs, 25 lbs, and 50 lbs), and screwing (at elbow height, kneeling, and overhead). Both the datasets consist of 38 features (3-acceleration, 3-gyroscope, and 32-EMG). Once the datasets were prepared, the ML-based classifiers were built with different sensor feature combinations. As explained in Experiment IV, the ten most used ML-based classifier models were built for three sensor data combinations for both datasets. The finely tuned ML-based classifiers were evaluated using 10-fold cross-validation, and the accuracy of the classifiers was combined across all the sensor feature combinations for both the datasets.

The classification involves identifying a set of classes using the input features. The performance of a classification algorithm is evaluated using metrics such as accuracy, recall, precision, F1 score, and kappa. In order to define these metrics, one needs to understand the terms true positives (TP), true negative (TN), false positive (FP), and false-negative (FN). The classification accuracy is the ratio of correct predictions (TP + TN) to the total number of predictions (TP + TN + FP + FN). Precision measures the number of correct positive predictions, which is the ratio of true positives (TP) to total positive predictions (TP + FP). In contrast, recall is the measure of the number of correct positive predictions out of all the positive predictions, which are the ratio of true positives to true positives (TP) and false-negatives (FN). F1 score is the weighted average of precision and recall, as shown in Equation (9) [60]. Cohen’s kappa value measures the agreement between the predicted and actual labels. Apart from these metrics, the performance of the classifier on individual classes was assessed by using a confusion matrix.

(9) $$F1\;\mathrm{Score}=2\times \frac{\mathrm{Precision}\times \mathrm{Recall}}{\mathrm{Precision}+\mathrm{Recall}}$$

## 3. Results

### 3.1. Forearm EMG and IMU Data Quality for “At-Rest” and “In-Motion” Activities

**Figure 4:**
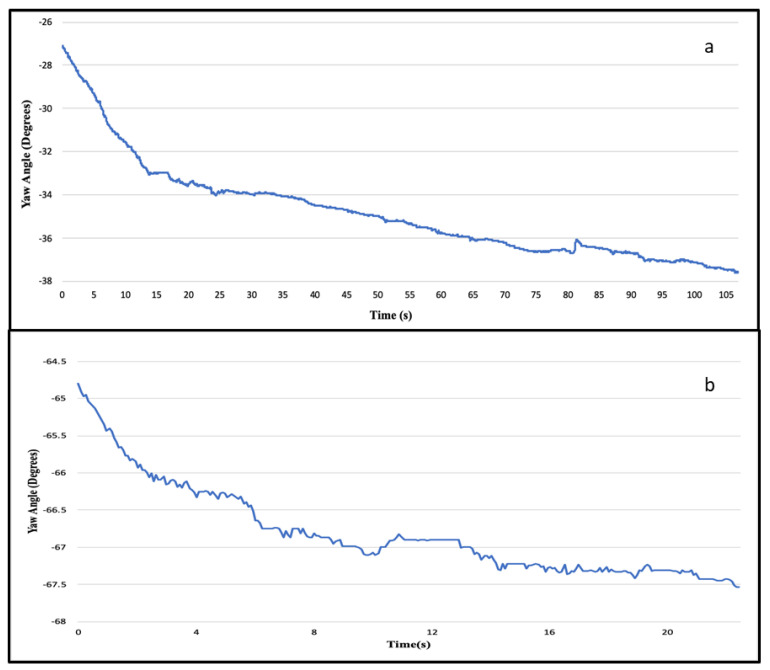
Evolution of yaw angle of armband sensor while (**a**) stationary on the body and (**b**) lying on the floor.

Firstly, the EMG and IMU data quality of the armband sensors were compared with conventional EMG (FREEEMG) and IMU (Yost) using standard deviation and signal to noise ratio. Table 1 shows the standard deviation (noise level) and SNR (signal quality) for accelerometer, gyroscope, and EMG for both conventional and armband sensor for at-rest and in-motion activities. The at-rest activities include stationary on the body for the EMG and stationary on the floor for IMU. For at-rest and in-motion activities, the noise levels in acceleration and gyroscope data of the armband sensor are comparable to a conventional sensor. The SNR values are higher in the case of armband acceleration data compared to the conventional sensor for both at-rest and in-motion activities. Whereas, the SNR values of gyroscope and EMG armband data are comparable to conventional sensors (Table 1). However, the signal quality measured as SNR is better in armband data compared to conventional sensors for both EMG and IMU (Table 1). Secondly, the noise level and data quality were compared between the indoor and outdoor environments. The results show that the noise level slightly increased in case of gyroscope (SD_Indoor_ = 0.121, and SD_Outdoor_ = 0.138) and EMG (SD_Indoor_ = 3.006, and SD_Outdoor_ = 2.974) data for outdoor environment (Table 2). However, the signal quality is comparably the same for both the environments (Table 2). Thirdly, two different armband sensors under same conditions have similar noise level and data quality for acceleration (SD_1_ = 0.002, SNR_1_ = 514.120; SD_2_ = 0.002, SNR_2_ = 515.192), gyroscope (SD_1_ = 0.121, SNR_1_ = 1.325; SD_2_ = 0.138, SNR_2_ = 1.469) and EMG (SD_1_ = 3.006, SNR_1_ = 0.865; SD_2_ = 2.947, SNR_2_ = 0.881) data (Table 3). The acceleration, gyroscope, and EMG data of stationary on the body were assessed for potential confounding factors, as shown in Table 4. The results show that the noise level in the acceleration is almost similar for all the factors; however, slightly affected in the presence of a communication device (Table 4). The noise level in gyroscope and EMG data have slightly increased in the presence of other sensor and power tools, respectively. However, the data quality of gyroscope and EMG data is similar in the presence and absence of confounding factors (Table 4). Finally, the rotational drift was determined by observing the evolution of the yaw angle for the data collected during the stationary on the body and Myo lying on the floor. Figure 4 shows the evolution of a yaw angle for 80 s of a stationary experiment. The results indicated that there was 0.13 deg/s drift initially and it reached a steady orientation when the armband was stationary on the body (Figure 4a). Whereas in the case of armband lying on the floor, the yaw angle drifts at a rate of 0.17 deg/s before it reached steady orientation, as shown in Figure 4b. Besides, it can be observed that the rotational drift was reduced considerably when worn on the body compared to the armband lying on the floor. Furthermore, the drift was higher in the initial frames and reached steady orientation in a few seconds. Therefore, a settling time of two minutes was considered to prevent rotational drift.

**Figure 5:**
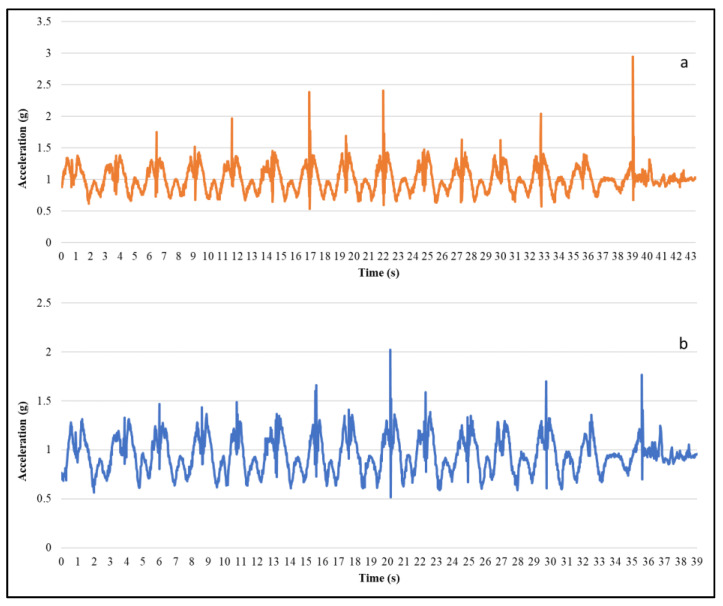
Acceleration magnitude while lifting using (**a**) conventional sensor and (**b**) armband sensor.

**Figure 6:**
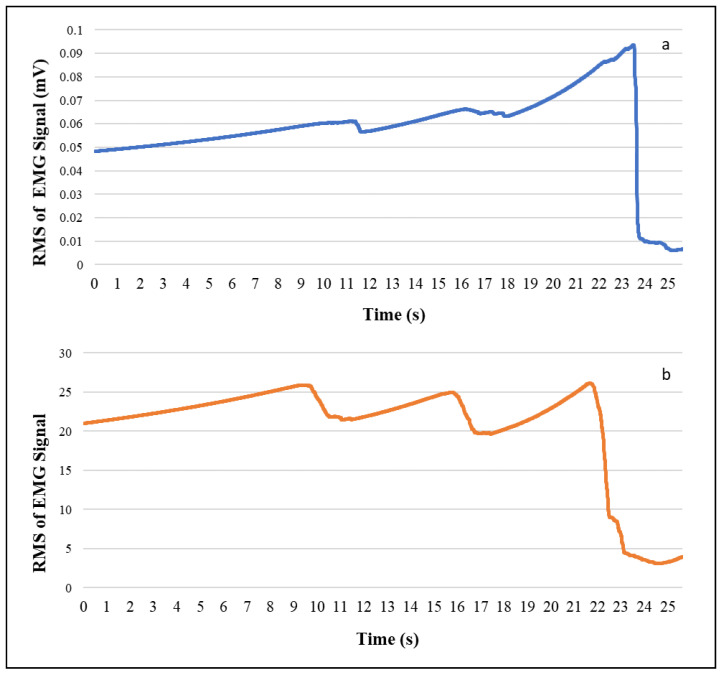
EMG RMS plots for lifting activity using (**a**) conventional EMG sensor and (**b**) armband sensor.

Further, a qualitative comparison was performed by inspecting the in-motion activity data from the armband and conventional sensors. The acceleration and EMG data of lifting activity of armband and conventional sensor wore at the same time was plotted in Figure 5 and Figure 6, respectively. In Figure 5, the acceleration magnitude was compared for both the sensors, and it is evident that the acceleration data pattern is similar to the conventional IMU sensor. In Figure 6, the root mean square (RMS) of EMG channel-4 was compared with conventional EMG RMS, which shows that they follow a similar trend. Moreover, the Myo armband can capture more detailed information compared to a single FREEEMG sensor.

### 3.2. Effect of Sensor Position on Forearm EMG and IMU Data Quality

**Figure 7:**
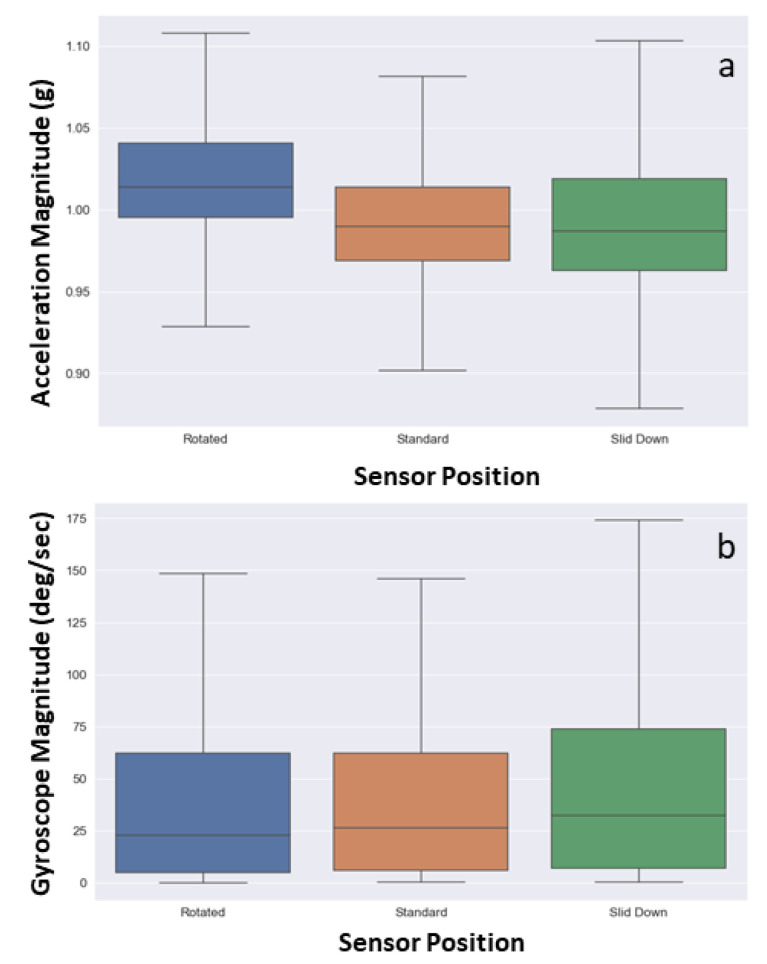
Comparison of (**a**) acceleration and (**b**) gyroscope magnitude for three (rotated, standard, and slid) sensor positions.

**Figure 8:**
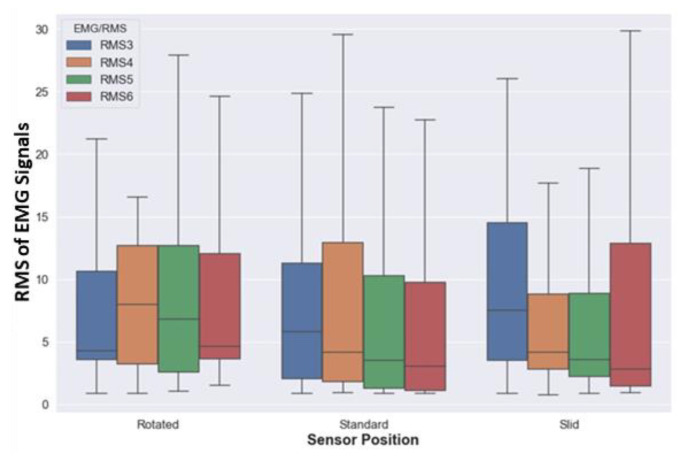
Comparison of RMS values of EMG-3, 4, 5, and 6 channels for three (rotated, standard, and slid) sensor positions.

The effect of three sensor positions, such as “rotated,” “standard,” and “slid down” are compared for lifting activity. Figure 7a,b shows the acceleration and gyroscope magnitude for three positions, the range of magnitude and median is the same for all the three positions, and this shows that the IMU data of the forearm is almost the same irrespective of the armband position; whereas the RMS plots of EMG vary for different sensor positions, as shown in Figure 8. When the armband is rotated by one sensor, the channel-5 takes the position of channel-4, and channel-6 takes the position of channel-5. Similarly, the RMS plot of EMG-5 and EMG-6 in rotated positions are similar to the RMS plot of EMG-4 and EMG-5 in standard positions, respectively. Whereas the RMS plots in slid down position have a lower magnitude range compared to the standard position due to lesser contact with the muscle in slid position.

### 3.3. Reliability of Forearm EMG and IMU Data of Construction Activities

The forearm acceleration, gyroscope, and EMG data from eight participants while performing construction activities such as screwing, wrenching, lifting, carrying, and at-rest was assessed for trial-to-trial and day-to-day reliability using the ICC test. Table 5, Table 6 and Table 7 summarize the test-retest reliability evaluation of accelerometer, gyroscope, and EMG measurements. For each activity, the mean and standard deviation of the measurements is the average of three trials (test mean (SD)) for each day. The average ICC value of three trials at a 95% confidence interval (CI), SEM%, and SDD% for all five activities for both days are shown in Table 5, Table 6 and Table 7. For acceleration measurements of both the days, the average ICC values range from 0.844 to 0.995 for all five activities (Table 5). Similarly, for gyroscope and EMG, the values range from 0.839 to 0.987 and 0.864 to 0.988, respectively (Table 6 and Table 7). The results from Table 5, Table 6 and Table 7 indicate excellent relative reliability between trials of acceleration, gyroscope, and EMG measurements for all five activities for both days. Moreover, SEM% for all the activities for acceleration, gyroscope, and EMG measurements is below 10%, which indicates excellent absolute reliability between trials for both the days. The SDD% for all activities for both the days ranges from 0.098% to 0.669% for acceleration, 5.953% to 32.225% for gyroscope, and 6.709% to 29.130% for EMG.

The day-to-day reliability assessment for accelerometer measurements shows that the ICC value is greater than 0.75, and SEM% is below 10% for all the activities, which indicates an excellent relative and absolute reliability for all activities (Table 8). For gyroscope, the ICC values are greater than 0.75 except for lifting activity (ICC = 0.724), which indicates excellent relative reliability of gyroscope data except for lifting. Whereas for the absolute reliability, SEM% values are below 10% except for stationary on the body (SEM% = 11.36%) and screwing (SEM% = 16.322%) activity. For EMG measurements, the ICC values are greater than 0.75 for all the activities indicating excellent relative reliability. Whereas the SEM% is slightly above 10% except for lifting activity (SEM% = 7.75%). The SDD% values range from between 14.48% to 31.48% and 24.49% to 39.89% for gyroscope and EMG measurements, respectively. The higher SDD% values of gyroscope and EMG suggest that caution should be taken when using gyroscope and EMG measurements for activity recognition because the change in the measurements might be due to error. Therefore, later experiments investigate if the data quality and reliability of the armband data are sufficient to yield accurate and reliable activity classification results.

### 3.4. Validating the Classifier Performance on Day-1 and Day-2 Dataset

Table 9 and Table 10 present the classification performance results of the classifiers built using Day-1 and Day-2 datasets. The performance of both classifiers was evaluated using overall accuracy, recall, precision, F1 score, and kappa, as shown in Table 9 and Table 10. The best classification performance was obtained for random forest for both Day-1 (accuracy—96.48%) and Day-2 datasets (accuracy—96.48%). Further, the random forest classifier was used to assess performance between the classes using the confusion matrix and class report, as shown in Table 11 and Table 12. The recall values above 90% for both the classifiers show that a specific activity can be predicted with less false positive values. The F1 score demonstrated high overall performance for stationary on the body, carrying, lifting, screwing, and with the lowest for wrenching (93.2% and 94.9%) for both the classifiers (Table 11 and Table 12). Finally, the association between the actual activities and the predicted classes was measured with Cohen’s kappa coefficient, and the values indicate strong agreement with the reality in both Day-1 (95.6% ± 0.003) and Day-2 (96.73% ± 0.0019) classifiers (Table 11 and Table 12).

### 3.5. Reliability of Classification Results

The classification results obtained using the classifiers of Day-1 and Day-2 were further analyzed for reliability using paired *t*-test on overall accuracy. A paired *t*-test (*p* = 0.63) at a significance level of 0.05 shows that their no significant difference between the accuracies of both the Day-1 and Day-2 classifier. The difference between the overall accuracy of Day-1 and Day-2 random forest classifier is 0.15%.

### 3.6. Effect of Lifting Weight on Classification Results

**Figure 9:**
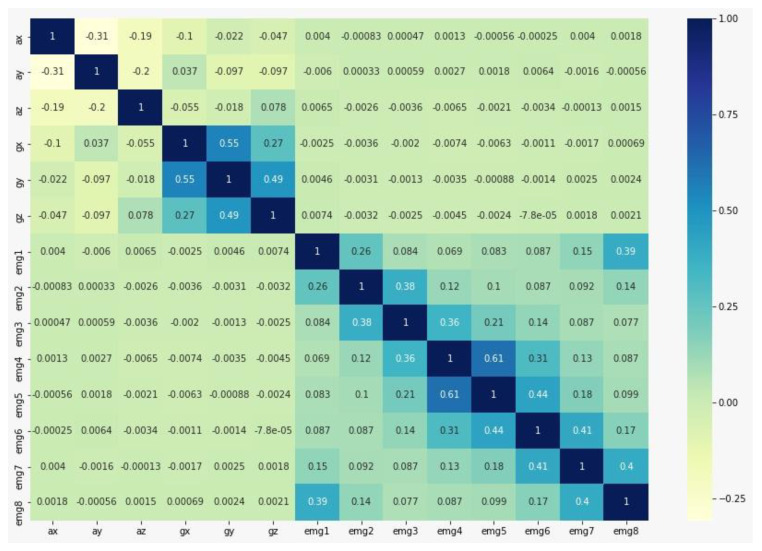
Correlation of features for three (10 lbs, 25 lbs, and 50 lbs) lifting weights.

The ten most common classification algorithms’ performances were analyzed on the lifting different weights dataset. Table 13 shows the accuracy, recall, precision, F1 score, and kappa values of all the classifiers. The random forest classifier showed the best performance in classifying three different weights with an overall accuracy of 83.89%, recall value of 84.06%, and kappa value of 75.82%. The results indicate that using the forearm EMG and IMU data, the random forest classifier can classify all three weights at 83.89% accuracy. Further, the confusion matrix and class report show that the high overall performance for Lift10 (F1 score = 91%) activity followed by Lift25 (F1 score = 80%) and Lift50 (F1 score = 77%) (Table 14). The results confirm that the forearm EMG and IMU data can not only classify lifting activity but is also able to detect the weight. In addition, the correlation of raw features shows that the gyroscope and EMG features are highly correlated compared to accelerometer features (Figure 9). Therefore, it can be concluded that the gyroscope and EMG features provide an opportunity to classify different weights of lifting activity.

### 3.7. Comparison of Activity Classification Performance for Different Sensor Combinations

The comparison of overall accuracies for different sensor combinations is shown in Table 15. For the controlled dataset, the EMG + IMU and IMU alone are better compared to EMG. The classification accuracy is higher for EMG + IMU in the case of random forest, SVM, naïve Bayes, and MLP; whereas, the classification accuracy is higher for IMU alone in the case of KNN, logistic, LDA, QDA, and Xgboost. However, except for KNN and MLP, the accuracy is not significantly different for EMG + IMU and IMU alone; whereas for the uncontrolled activity dataset, the accuracy is significantly higher for EMG + IMU compared to IMU and EMG alone except in the case of KNN. For the KNN classifier, the IMU alone has higher accuracy compared to EMG + IMU. However, the highest classification accuracy (98.13%) for nine activities with various intensities was obtained for the EMG + IMU feature combination. The combination of EMG and IMU features yields higher accuracy compared to individual sensor data for complex activities.

## 4. Discussion

In this study, the data quality of low-cost forearm based wearable sensors were explored by comparing the standard deviation and signal to noise ratio of the armband sensor and the conventional sensor for at-rest and in-motion activities. The noise levels in the armband acceleration data (SD = 0.002) when lying on the floor are comparable to the high precision conventional IMU sensor (SD = 0.003), which is in agreement with the previous study (SD = 0.0019) [25]. Similarly, the noise levels in the acceleration and gyroscope data for in-motion activities are comparable to conventional sensors. Besides, the signal quality of armband sensor data is higher compared to the conventional sensor, which shows that the armband sensor is less sensitive compared to high precision and high-frequency sensors. Moreover, the data quality test in the presence of confounding factors also proves that the armband data is not affected much by the confounding factors, environment, and inter-device variability. Drift is one of the most common issues of IMU when used to estimate position and orientation [26]. The rotational drift of the armband sensor was assessed by observing the evolution of the yaw angle for at-rest activities. The yaw angle drifts at a rate of 0.17 deg/s before it reaches the steady orientation, which is in agreement with a previous study [61]. This experiment proves that the drift reduced when the Myo was worn on the body compared to lying on the floor. Moreover, the rotational drift was highest in the initial frames and reached a steady state in a few seconds. Similar to the other studies [25,62], the in-motion (i.e., lifting) activity data of the armband and the conventional sensor was visually compared since the quantitative comparison of both sensor signal data would not be appropriate. For the comparison of EMG and accelerometer signals, RMS and absolute magnitude plots were considered, as shown in Figure 5 and Figure 6. The result shows that the armband data and conventional sensor both pick the same peaks and follow a similar trend for lifting activity. The qualitative assessment of armband sensor position on EMG and IMU data quality shows that accelerometer and gyroscope data is almost similar for three (rotated, standard, and slid down) sensor positions. A previous study [63] reported similar results where the classification accuracy using accelerometer data at different sensor positions made no significant difference. However, the EMG data for three armband positions are significantly different, which conforms with the fact that the IMU sensor captures the motion of the forearm, whereas the EMG signal depends on the muscle contact.

The study assessed the relative and absolute reliability of forearm EMG and IMU data of construction activities. The test-retest evaluation of accelerometer data indicated an excellent trial-to-trial (ICC = 0.844 to 0.995 and SEM% = 0.087% to 0.258%) and day-to-day (ICC = 0.824 to 0.881 and SEM% = 0.245% to 0.526%) relative and absolute reliability for all the activities as shown in Table 5. Whereas for the gyroscope data, an excellent relative reliability was observed for trial-to-trial (ICC = 0.824 to 0.987) and day-to-day (ICC = 0.801 to 0.844) except for lifting where ICC = 0.724 (Table 6 and Table 7). The absolute reliability of gyroscope data for day-to-day was slightly greater than 10% ranging from 5.224% to 16.322%. The EMG data has shown excellent relative (ICC = 0.864 to 0.988) and absolute (SEM% = 2.420% to 10.509%) reliability between trials but the absolute reliability between the days (SEM% = 7.75% to 16.21%) is slightly greater than 10% (Table 8). Overall, the results show that armband sensor data (acceleration, gyroscope, and EMG) exhibited excellent relative reliability between trials and days, which indicates a strong correlation of the repeated measurements. Furthermore, the armband sensor data exhibited excellent absolute reliability between the trails and moderate absolute reliability between days, which is indicated with a slight increase in SEM% and SDD%. As shown in Equations (6) and (8), SEM% and SDD% are directly correlated to the ratio of SD and mean of the measurements. The higher SEM% and SDD% between days are due to the larger SD to mean ratio. Further investigation was performed to determine if the armband data obtained at this level of reliability is sufficient to yield accurate and reliable activity classification results.

The ML-based classification results using both days’ datasets show that the forearm EMG, acceleration, and gyroscope features are capable of classifying activities involving different body parts such as wrist, forearm, and whole-body and various motions such as repetitive motion, repeated impulsive motion, and free motion with high accuracy (Day-1_accuracy_ = 96.48% ± 0.0024 and Day-2_accuracy_ = 96.33% ± 0.0022). Furthermore, the overall classification accuracy of 98.13% achieved for nine uncontrolled activity datasets shows that the model is capable of recognizing activity with different intensities, which is one of the limitations of current construction activity recognition models [10,16,20]. The accuracy of proposed activity recognition models using EMG and IMU forearm data (Accuracy_EMG + IMU_ = 98.13%) is higher than previously published construction activity recognition models such as carpentry activities (91%) [14], fall identification (94%) [15], manual material handling activities (90.74%) [11], ironworker activities (94.83%, 92.98%) [9,17], and bricklaying activities (88.1%) [20].

Moreover, the reliability assessment of classification results using Day-1 and Day-2 classifiers showed that there exists excellent reliability of classification results using the forearm EMG and IMU features. Later, the forearm EMG and IMU data were used to classify different weights of lifting activity, which is useful for various construction applications. The results showed that the overall classification accuracy of three classes (Lift10, Lift25, and Lift50) is 83.89% (0.0051), which is higher than the accuracy obtained by Ho, et al. [64] (77.1%) in classifying barbell weights from 20 to 70 lbs using forearm EMG features. Moreover, for three lifting weights, the gyroscope and EMG features are highly correlated, which contributed to higher classification accuracy. The comparison of classification performance for different sensor combinations on controlled (Accuracy_EMG + IMU_ = 96.21%, Accuracy_IMU_ = 94.65%, Accuracy_EMG_ = 44.97%) and uncontrolled (Accuracy_EMG + IMU_ = 98.21%, Accuracy_IMU_ = 84.80%, Accuracy_EMG_ = 47.60%) dataset showed that the highest accuracy is obtained in case of EMG + IMU which is in agreement with the previous studies on forearm gym activities (Accuracy_EMG + IMU_ = 71.6%, Accuracy_IMU_ = 67.8%, Accuracy_EMG_ = 20.7%) [38], forearm manufacturing activities (Accuracy_EMG + IMU_ = 87.4%, Accuracy_IMU_ = 85.0%, Accuracy_EMG_ = 50.7%) [41], and gym exercises (Accuracy_EMG + IMU_ = 84.2%, Accuracy_IMU_ = 77.7%, Accuracy_EMG_ = 85.2%) [47]. Further, the increase in classification accuracy due to combined features show that the gyroscope and EMG features obtained at higher SEM% and SDD% are suitable for activity classification. The fusion of forearm muscle activity (EMG) and kinematic (IMU) data have resulted in the highest classification accuracy for a greater number of complex activities with different intensities. The advantage of using an armband sensor is that both forearm muscle activity and motion data are obtained from the single device and avoids the use of multiple sensors that obstructs construction work.

Some of the limitations of the study worth mentioning are that the data quality of the sensor data was assessed only on at-rest activities. All the in-motion activities were performed in residential settings by participants with little to no construction experience. All the participants in this study were right-handed and male. In addition to acceleration, gyroscope, and EMG data, the armband sensor provides orientation quaternion and Euler angles of the forearm. However, the orientation angles were not assessed for reliability in this study. Moreover, one can consider performing validity assessment for forearm EMG and IMU data of armband sensor.

## 5. Conclusions

The current study assessed the data quality and reliability of forearm EMG and IMU data from a low-cost wearable sensor for activity classification. In order to achieve the objective, the whole study was divided into seven experiments. From the first experiment, the data was inferred that the armband sensor data is comparable to conventional EMG and IMU data. Moreover, there was a very minimal effect of environment, confounding factors (communication device, power tools, other sensors, and smartwatches), and inter-device variability. Secondly, a qualitative comparison was performed to understand the effect of armband position on forearm EMG and IMU data, and it was concluded that the armband position does not affect IMU data, but EMG data was affected due to the sensor position. Thirdly, the trial-to-trial and day-to-day reliability of acceleration, gyroscope, and EMG data were assessed for five construction activities. The results conclude that the forearm IMU and EMG data for all five activities have excellent relative and absolute reliability between the trials, and between the days except for EMG data between the days has SEM% slightly higher than 10%. Next, the EMG and IMU data for both days was used to build and evaluate building ML-based activity classification models. The most common classification models were compared for the performance on the Day-1 and Day-2 datasets. The random forest classification algorithm showed the best performance on both the datasets. The reliability test on the classification results of both the classifiers confirmed that the classification results are high reliability with minimal change inaccuracies for both the days. The effect of lifting weight on classification performance was assessed, which concluded that the forearm EMG and IMU data could classify three different weights. Further, it was observed that a strong correlation in gyroscope and EMG features exists compared to accelerometer data for three classes. Finally, the comparison of classification performance for different sensor combinations showed that the forearm muscle activity and motion data fusion yield higher classification accuracy for construction activities with various intensities. The armband data is highly reliable, and the scientific evaluation of the armband sensor builds trustworthiness among researchers, policymakers, stakeholders, and customers to use the sensor for various applications. The data quality and reliability assessment of armband sensors show that the quality of muscle and motion-sensing data is sufficient for various construction applications related to construction skill training, safety training, and monitoring. Moreover, the classification results of the study conclude that the forearm-based EMG and IMU data can be used to generate reliable construction activity, recognition models.

## Figures and Tables

**Table 1:** Comparison of EMG and IMU data quality of Myo armband and conventional sensors.

	Accelerometer (Units of g)				Gyroscope (rad/s)				EMG			
	Myo		Conv.		Myo		Conv.		Myo		Conv.	
	SD	SNR	SD	SNR	SD	SNR	SD	SNR	SD	SNR	SD	SNR
At-rest Activities	0.00	514.12	0.00	340.64	0.00	1.32	0.04	0.32	3.01	0.87	0.00	0.83
Screwing	0.02	60.42	0.03	35.30	0.31	0.52	0.62	0.43	4.39	0.78	0.02	0.67
Wrenching	0.03	37.10	0.04	25.88	0.39	0.66	0.40	0.71	5.64	0.70	0.02	0.67
Lifting	0.13	8.02	0.15	6.96	0.95	0.90	0.97	0.89	19.50	0.49	0.07	0.41
Carrying	0.06	16.07	0.07	15.35	0.45	1.22	0.50	1.16	11.25	0.70	0.01	0.66

**Table 2:** Comparison of EMG and IMU data quality between indoor and outdoor for at-rest activity.

	Indoor		Outdoor	
	Std. Dev.	SNR	Std. Dev.	SNR
Accelerometer	0.002	514.120	0.002	495.712
Gyroscope	0.121	1.325	0.223	1.093
EMG	3.006	0.865	3.389	0.846

**Table 3:** Comparison of EMG and IMU data quality between two armband sensors for at-rest activity.

	Myo-1		Myo-2	
	Std. Dev.	SNR	Std. Dev.	SNR
Accelerometer	0.002	514.120	0.002	515.192
Gyroscope	0.121	1.325	0.138	1.469
EMG	3.006	0.865	2.974	0.881

**Table 4:** Effect of confounding factors on data quality for at-rest activity.

	Communication Device		Other Sensor		Power Tool		Smart Watch	
	Std. Dev.	SNR	Std. Dev.	SNR	Std. Dev.	SNR	Std. Dev.	SNR
Accelerometer	0.002	443.860	0.002	470.081	0.002	493.564	0.002	479.490
Gyroscope	0.100	1.588	0.166	1.157	0.133	1.147	0.111	1.255
EMG	3.270	0.851	3.100	0.855	4.610	0.830	3.030	0.855

**Table 5:** Accelerometer trial-to-trial reliability.

Day-1					Day-2			
	Test Mean (SD)	ICC (95% CI)	SEM%	SDD%	Test Mean (SD)	ICC (95% CI)	SEM%	SDD%
Stationary on the Body	0.97 (0.0048)	0.961 (0.943–0.990)	0.100%	0.285%	0.97 (0.00504)	0.995 (0.993–0.998)	0.035%	0.098%
Screwing	0.98 (0.0099)	0.965 (0.952–0.979)	0.190%	0.537%	0.98 (0.00931)	0.991 (0.988–0.995)	0.087%	0.245%
Wrenching	0.99 (0.0127)	0.978 (0.964–0.985)	0.192%	0.539%	0.99 (0.01227)	0.980 (0.969–0.995)	0.179%	0.490%
Lifting	1.00 (0.0103)	0.959 (0.952–0.963)	0.212%	0.585%	0.99 (0.00673)	0.923 (0.892–0.962)	0.203%	0.524%
Carrying	1.01 (0.0073)	0.888 (0.868–0.900)	0.245%	0.669%	1.01 (0.00551)	0.844 (0.779–0.931)	0.258%	0.598%

**Table 6:** Gyroscope trial-to-trial reliability assessment.

Day-1					Day-2			
	Test Mean (SD)	ICC (95% CI)	SEM%	SDD%	Test Mean (SD)	ICC (95% CI)	SEM%	SDD%
Stationary on the Body	0.575 (0.155)	0.921 (0.840–0.966)	7.981%	22.122%	0.581 (0.168)	0.839 (0.757–0.924)	11.626%	32.225%
Screwing	9.448 (3.850)	0.987 (0.986–0.987)	4.706%	13.043%	7.316 (1.900)	0.893 (0.873–0.930)	8.507%	23.581%
Wrenching	14.243 (3.291)	0.967 (0.954–0.988)	4.176%	11.577%	14.239 (2.538)	0.885 (0.854–0.935)	6.036%	16.731%
Lifting	46.043 (5.164)	0.963 (0.948–0.979)	2.148%	5.953%	45.945 (3.897)	0.824 (0.759–0.873)	3.562%	9.874%
Carrying	30.804 (3.960)	0.899 (0.864–0.921)	4.092%	11.344%	24.838 (5.609)	0.919 (0.880–0.939)	6.440%	17.851%

**Table 7:** EMG trial-to-trial reliability assessment.

Day-1					Day-2			
	Test Mean (SD)	ICC (95% CI)	SEM%	SDD%	Test Mean (SD)	ICC (95% CI)	SEM%	SDD%
Stationary on the Body	10.183 (4.678)	0.981 (0.963–0.995)	6.332%	17.552%	7.80 (1.163)	0.864 (0.817–0.917)	5.499%	15.242%
Screwing	23.622 (8.206)	0.946 (0.914–0.992)	8.048%	22.308%	27.32 (7.602)	0.983 (0.973–0.990)	3.628%	10.056%
Wrenching	31.719 (11.066)	0.988 (0.985–0.989)	3.874%	10.739%	30.84 (11.966)	0.983 (0.977–0.988)	5.108%	14.159%
Lifting	40.497 (4.963)	0.961 (0.946–0.976)	2.420%	6.709%	40.65 (8.490)	0.948 (0.918–0.979)	4.748%	13.160%
Carrying	42.326 (16.037)	0.949 (0.937–0.962)	8.557%	23.718%	35.77 (10.282)	0.866 (0.806–0.914)	10.509%	29.130%

**Table 8:** Accelerometer, gyroscope, and EMG day-to-day reliability assessment.

	Accelerometer			Gyroscope			EMG		
	Day-1 vs. Day-2			Day-1 vs. Day-2			Day-1 vs. Day-2		
	ICC	SEM%	SDD%	ICC	SEM%	SDD%	ICC	SEM%	SDD%
Stationary on the Body	0.82	0.24%	0.57%	0.80	11.36%	31.48%	0.92	12.57%	34.83%
Screwing	0.86	0.40%	0.97%	0.80	16.32%	45.24%	0.85	12.14%	33.65%
Wrenching	0.86	0.52%	1.26%	0.84	8.22%	22.79%	0.79	16.21%	44.92%
Lifting	0.86	0.35%	0.86%	0.72	5.22%	14.48%	0.82	7.75%	21.49%
Carrying	0.88	0.24%	0.59%	0.78	6.94%	19.25%	0.85	14.39%	39.89%

**Table 9:** ML-based classifier performance on Day-1 dataset.

Classifier	Accuracy	Recall	Precision	F1 Score	Kappa
Random Forest	96.48% (0.0024)	96.39% (0.0025)	96.49% (0.0024)	96.48% (0.0024)	95.60% (0.0030)
J48	94.30% (0.0017)	94.17% (0.0017)	94.78% (0.0016)	94.38% (0.0016)	96.33% (0.0022)
SVM	58.85% (0.0084)	57.59% (0.0086)	58.01% (0.0172)	54.31% (0.0165)	48.44% (0.0105)
Naïve Bayes	70.45% (0.0022)	69.87% (0.0021)	70.64% (0.0023)	70.06% (0.0023)	63.06% (0.0027)
KNN	79.43% (0.0024)	78.853% (0.0025)	79.55% (0.0025)	78.95% (0.0026)	74.25% (0.0030)
Logistic	61.64% (0.0044)	60.57% (0.0045)	64.28% (0.0052)	60.51% (0.0046)	51.96% (0.0055)
MLP	94.81% (0.0029)	94.67% (0.0029)	94.80% (0.0028)	94.80% (0.0028)	93.51% (0.0036)
LDA	59.11% (0.0037)	57.92% (0.0037)	63.11% (0.0081)	56.21% (0.0036)	48.76% (0.0046)
QDA	75.23% (0.0028)	74.65% (0.0029)	75.45% (0.0032)	74.86% (0.0031)	69.03% (0.0035)
Xgboost	93.42% (0.0029)	93.22% (0.0030)	93.50% (0.0028)	93.36% (0.0030)	91.77% (0.0037)

**Table 10:** ML-based classifier performance on Day-2 dataset.

Classifier	Accuracy	Recall	Precision	F1 Score	Kappa
Random Forest	97.43% (0.0015)	97.35% (0.0015)	97.44% (0.0015)	97.43% (0.0015)	96.73% (0.0019)
J48	96.01% (0.0014)	95.99% (0.0014)	96.24% (0.0013)	96.05% (0.0014)	95.02% (0.0017)
SVM	66.50% (0.0069)	65.64% (0.0072)	66.03% (0.0061)	63.69% (0.0090)	58.03% (0.0087)
Naïve Bayes	73.27% (0.0036)	72.80% (0.0037)	73.81% (0.0034)	72.93% (0.0034)	66.61% (0.0046)
KNN	90.98% (0.0015)	91.00% (0.0012)	90.90% (0.0012)	90.90% (0.0012)	88.72% (0.0015)
Logistic	68.31% (0.0046)	67.82% (0.0046)	69.55% (0.0044)	67.97% (0.0046)	60.34% (0.0057)
MLP	97.13% (0.0019)	97.04% (0.0019)	97.13% (0.0018)	97.13% (0.0018)	96.41% (0.0023)
LDA	68.43% (0.0054)	67.88% (0.0055)	71.43% (0.0054)	67.70% (0.0056)	60.45% (0.0068)
QDA	76.32% (0.0035)	75.90% (0.0036)	77.00% (0.0033)	75.79% (0.0033)	70.42% (0.0043)
Xgboost	96.51% (0.0012)	96.39% (0.0012)	96.50% (0.0012)	96.50% (0.0012)	95.64% (0.0015)

**Table 11:** Confusion matrix and class report of random forest classifier on Day-1 dataset.

	Predicted Class					
		Stationary on the Body	Screwing	Wrenching	Lifting	Carrying
**True Class**	**Stationary on the Body**	8953	27	2	0	0
	**Screwing**	13	8244	345	38	0
	**Wrenching**	10	409	8035	209	0
	**Lifting**	1	129	187	8131	97
	**Carrying**	0	1	5	64	9598
Overall Accuracy						96.48% (0.0024)
Precision		99.70%	93.60%	93.60%	96.30%	99.00%
Recall		99.70%	95.40%	92.80%	95.10%	99.30%
F1 Score		99.70%	94.50%	93.20%	95.70%	99.10%

**Table 12:** Confusion matrix and class report of random forest classifier on Day-2 dataset.

	Predicted Class					
		Stationary on the Body	Screwing	Wrenching	Lifting	Carrying
**True Class**	**Stationary on the Body**	11,857	25	0	0	0
	**Screwing**	14	10,941	210	75	0
	**Wrenching**	0	368	9949	220	0
	**Lifting**	0	75	247	11,184	59
	**Carrying**	0	21	32	47	11,653
Overall Accuracy						96.33% (0.0022)
Precision		99.90%	95.70%	95.30%	97.00%	100%
Recall		99.80%	97.30%	94.40%	96.70%	99%
F1 Score		99.80%	96.50%	94.90%	96.90%	99%

**Table 13:** Classifier performance on lifting different weights.

Classifier	Accuracy	Recall	Precision	F1 Score	Kappa
Random Forest	83.89% (0.0051)	83.71% (0.0053)	84.06% (0.0053)	83.93% (0.0052)	75.82% (0.0077)
J48	72.71% (0.0061)	72.73% (0.0061)	76.14% (0.0067)	73.05% (0.0060)	59.91% (0.0091)
SVM	54.31% (0.0079)	53.43% (0.0082)	53.26% (0.0163)	48.94% (0.0134)	31.00% (0.0123)
Naïve Bayes	43.21% (0.0080)	42.17% (0.0082)	43.86% (0.0169)	36.94% (0.0109)	13.61% (0.0123)
KNN	65.07% (0.0040)	64.47% (0.0003)	64.98% (0.0014)	63.91% (0.0001)	47.45% (0.0006)
Logistic	56.70% (0.0054)	55.96% (0.0055)	54.78% (0.0072)	53.57% (0.0062)	34.70% (0.0082)
MLP	71.838% (0.0043)	82.90% (0.0015)	83.00% (0.0054)	82.90% (0.0003)	31.14% (0.0093)
LDA	55.07% (0.0054)	54.35% (0.0056)	53.08% (0.0071)	52.15% (0.0060)	32.31% (0.0082)
QDA	45.69% (0.0069)	44.68% (0.0070)	46.11% (0.0104)	40.70% (0.0104)	17.50% (0.0106)
Xgboost	75.84% (0.0070)	75.58% (0.0072)	75.91% (0.0074)	75.81% (0.0072)	63.73% (0.0106)

**Table 14:** Confusion matrix and class report of random forest classifier on different lifting weights.

	Predicted Class			
		Lift10	Lift25	Lift50
**True Class**	**Lift10**	3622	221	242
	**Lift25**	94	3135	603
	**Lift50**	169	669	2894
Overall Accuracy				83.89% (0.0051)
Precision		93%	78%	77%
Recall		89%	82%	78%
F1 Score		91%	80%	77%

**Table 15:** Overall classification accuracy for different sensor combinations for controlled and uncontrolled datasets.

	Controlled Activity Dataset			Uncontrolled Activity Dataset		
Classifier	EMG + IMU	IMU	EMG	EMG + IMU	IMU	EMG
Random Forest	96.21%	94.65%	44.97%	98.13%	84.80%	47.60%
J48	94.94%	95.33%	48.54%	96.55%	78.55%	30.83%
SVM	73.23%	73.33%	21.21%	96.55%	48.39%	14.19%
Naïve Bayes	71.40%	69.39%	45.95%	82.52%	54.79%	23.05%
KNN	86.16%	96.95%	45.58%	71.03%	84.62%	29.83%
Logistic	64.65%	64.69%	18.86%	88.76%	45.63%	14.11%
MLP	90.87%	81.99%	52.27%	90.82%	62.50%	37.51%
LDA	62.78%	62.87%	18.87%	88.26%	26.57%	14.19%
QDA	74.79%	75.73%	46.44%	62.33%	52.66%	29.24%
Xgboost	41.53%	41.53%	27.51%	85.52%	21.72%	15.70%
